# Is intra-bladder pressure measurement a reliable indicator for raised intra-abdominal pressure? A prospective comparative study

**DOI:** 10.1186/s12871-018-0539-z

**Published:** 2018-06-19

**Authors:** Abdulla Ahmed Al-Abassi, Azan Saleh Al Saadi, Faisal Ahmed

**Affiliations:** 1 Department of Surgery, Saqr Hospital, Ras Al Khaimah, United Arab Emirates; 2 Department of Urology, Saqr Hospital, Ras Al Khaimah, United Arab Emirates

**Keywords:** Intra-abdominal pressure, Urinary bladder pressure, Intra-vesical pressure, Compartment syndrome, Intra-abdominal hypertension

## Abstract

**Background:**

Intra-abdominal pressure (IAP) can be measured by several indirect methods; however, the urinary bladder is largely preferred. The aim of this study was to compare intra-bladder pressure (IBP) at different levels of IAPs and assess its reliability as an indirect method for IAP measurement.

**Methods:**

We compared IBP with IAP in twenty-one patients undergoing laparoscopic cholecystectomy under general anesthesia. Measurements were recorded at increasing levels of insufflation pressures to approximately 22 mmHg. Pearson’s correlation coefficient was calculated to establish the relationship between the two pressure measurements and Bland-Altman analysis was used to assess the limits of agreement between the two methods of measurements.

**Results:**

The urinary bladder pressures reflected well the pressures in the abdominal cavity. Pearson correlation coefficient showed a good correlation between the two measurement techniques (*r* = 0.966, *p* < 0.0001) and Bland-Altman analysis indicated that the 95% limits of agreement between the two methods ranged from − 2.83 to 2.64. This range is accepted both clinically and according to the recommendations of the World Society of Abdominal Compartment Syndrome (WSACS).

**Conclusion:**

Our study showed that IBP measurement is a simple, minimally invasive method that may reliably estimates IAP in patients placed in supine position. Measurements for pressures higher than 12 mmHg may be less reliable. When applied clinically, this should alert the clinician to take safety measures to avoid abdominal compartment syndrome (ACS).

## Background

Intra-abdominal Hypertension (IAH) is defined as an intra-abdominal pressure (IAP) equal to or above 12 mmHg and abdominal compartment syndrome (ACS) is defined as an IAP above 20 mmHg with evidence of organ dysfunction/failure [1]. Both levels of increased intra-abdominal tension are known to causes significant morbidity and mortality among critically ill patients. This includes acute renal failure, pulmonary impairment, and reduced blood flow to the gastro-intestinal organs [1, 2]. In addition, increased IAP can cause elevation of the diaphragm with consequent increase in intra-thoracic pressure, which has a major effect on pulmonary compliance [3]. The high intra-thoracic pressure may interfere with the cerebral venous return via the jugular venous system leading to intra-cranial congestion and brain dysfunction [4].

Clinically, significant intra-abdominal hypertension may be present in the absence of abdominal distension [5]. Therefore, increased IAP is commonly measured by recording urinary bladder pressure (IBP). Kron et al. were the first to describe this technique in 1984 [2]. Later, Cheatham and Safcsak revised this technique by creating a system that was entirely closed, thus making the process of serial bedside IAP determinations easier, safer, and unlikely to introduce bladder infection [6].

The reliability of the urinary bladder as an indirect tool for measuring the IAP clinically is not agreed upon and human studies correlating IAP and IBP are scarce and far from being accurate and reproducible [5]. Supporting evidence comes from Fusco et al. [6] who found that the IBP closely approximates IAP in 37 patients. On the other hand, Johna et al. [4] found that IBP did not reflect the actual IAP in a clinical study of 21 patients undergoing laparoscopic cholecystectomy. Yol et al. [7] found that the IBP correlated well with the insufflator pressure in 40 patients undergoing laparoscopic cholecystectomy. However, the last two studies lack statistical accuracy, and their methodologically has been criticized [5, 8]. Moreover, techniques of simulating increased IAP via instillation of normal saline or insufflation of carbon dioxide as in laparoscopy are far from being an ideal environment for extrapolation to critically ill patients.

Our study aimed to determine the reliability of IBP measurement as an indirect measure of IAP. We conducted a prospective study in 21 patients undergoing laparoscopic cholecystectomy. We measured the IAP and IBP by gradually increasing the insufflator pressure to normal, then to high IAP of approximately 22 mmHg for a very short period to avoid any deleterious effects on patients’ hemodynamics. The use of such high pressure is based on the recommendations of the WSACS [8] and the study of Fusco et al. [6] who used insufflation pressures up to 25 mmHg without risking patient safety. We took into consideration the recommendations suggested by Malbrain M. [5] like proper positioning of the patient, the ideal fluid volume instilled into the bladder, proper positioning of the pressure transducers, and avoiding IAP measurement via Veress needles. In addition, statistical recommendations of the World Society of Abdominal Compartment Syndrome (WSACS) have generally been fulfilled [8, 9].

## Methods

This study was conducted at Saqr Hospital, Ras Al Khaimah, United Arab Emirates, in accordance with the study protocol approved by the hospital research ethics committee. All patients consented for participation in the study and agreed for publishing study results in medical journals.

Twenty-one patients aged 21–54 years and undergoing laparoscopic cholecystectomy between January and August 2007 were recruited for the study. Consenting for IBP and IAP measurements under general anesthesia were obtained. Patients with urinary symptoms, urinary bladder diseases, history of heart disease or chronic pulmonary disease were excluded. Pregnant women and children were also excluded.

Bladder pressure measurements were conducted by using DantecMenuet equipment (M 247) and fluid-filled lines with external pressure transducers. An 8 F double-lumen pressure catheter was introduced into the urinary bladder transurethral under sterile conditions. One lumen was used to evacuate the bladder and infuse 50 ml of normal saline at room temperature. The other lumen was used for pressure measurement. A 10 F single-lumen pressure catheter with a balloon tip connected to a pressure transducer was then introduced through the epigastric port and the balloon tip is placed intraperitoneally under direct vision. The catheter has a 3-way extension through which the balloon is filled with normal saline and after evacuation of air the fluid is retained in the syringe. Once the catheter is placed in the abdomen the fluid is flushed back into the balloon. The transducer tubings are infused with normal saline to evacuate the air and calibrated to zero pressure before connecting to the urethral pressure catheter.

All pressure measurements were performed with the patients lying in supine position under general anesthesia before starting laparoscopic cholecystectomy. The level of the symphysis pubis has been chosen for calibrating the pressure transducers for its simplicity to localize, taking into consideration that the mid axillary line is difficult to identify with the patient draped under general anesthesia. Zero balancing (calibrating the transducer’s output signal at zero pressure) was performed at atmospheric pressure with a stabilizing period of about 3 min before taking pressure measurements. Once the connections are made, to avoid any artifacts, the pressure lumen catheters are flushed with a minimal amount of saline through the transducer channel to avoid any air bubble or gel interruption.

The baseline IAP and IBP measurements are pressures recorded when no air insufflations applied to the abdomen. The two pressure measurements were then simultaneously recorded by stepwise increment of IAP by increasing the insufflation pressure gradually to approximately 22 mmHg, as shown on the laparoscopy CO2 insufflator. The readings are detected by DantecMenuet equipment and documented internally as the pressure goes up. We allowed a short period of stabilization before each reading. The number of readings and the level of pressures at each reading measured were not predefined and are not the same for each patient. Intra-peritoneal cavity then gradually deflated, the recording measurements took approximately 10–15 min, the catheters were removed and laparoscopic cholecystectomy was performed. The data collected from the system memory was transmitted to a computer and saved for later analysis.

### Statistics

Pearson’s correlation coefficient (r) was calculated to assess the strength of relation between IAP and IBP measurements. For quantitative analysis. Agreement between the two methods of measurements was assessed and plotted using Lin’ concordance correlation coefficient (LCCC) [10] and Bland-Altman method [11]. Internal consistency for all measurements was assessed using Cronbach’s α coefficient [12] with cut-offs of .8 denoting good and .9 denoting excellent reliability. Although not universally agreed upon, we used the descriptive scale suggested by McBride [13] to interpret the LCCC for strength of agreement. In Bland-Altman method, differences between the two methods are plotted against their averages. Horizontal lines indicate the mean difference (bias) and ± 1.96 × the standard deviation. This is useful to calculate and visualize the trend of the differences.

Statistical analyses and figures produced for all pairs of data were performed using “Medcalc” version 12.7 (Medcalc Software, Mariakerke, Belgium) and Statistical Package for the Social Sciences (SPSS, version 21; IBM, New York, NY, USA). SPSS was also used to convert all pressure measurements, which were recorded in cmH_2_O, to mmHg using the SPSS ‘transform’ tab before data analysis (conversion coefficient = 0.735).

## Results

Twenty-one patients aged 21–54 (Average 31.8) years underwent laparoscopic cholecystectomy. Twenty were female patients (95%). All 21 patients completed their surgery without any consequences from our intervention and had uneventful postoperative period.

Three hundred and eighty eight simultaneous pressure measurements from all 21 patients were recorded at varying IAPs. Out of these measurements, 20.9% were above 12 mmHg (IAH) and 3.1% were above 20 mmHg (grade III IAH).

When the IAP was elevated by intra-peritoneal carbon dioxide (CO_2_) insufflation to approximately 22 mmHg, the IBP showed parallel increments to about the same levels. Similarly, during gradual deflation of CO_2_ and reduction of the IAP to about 0 mmHg, IBP showed a comparable decrease to about the same level as the IAP.

Pearson correlation coefficient showed a strong correlation between the two measurements (*r* = 0.966, *p* < 0.0001) (Table 1).

**Table 1:** Lin’s Concordance & Pearson Correlation Coefficients between the two methods: IAP & IBP for all measures (IAP = 0–22 mmHg) Versus High IAP (IAP > 12 mmHg)

Variable Y	Intra Abdominal Pressure (IAP = 0–22 mmHg)	Intra Abdominal Pressure (IAP > 12 mmHg)
Variable X	Intra Bladder Pressure (IBP with IAP = 0–22 mmHg)	Intra Bladder Pressure (IBP with IAP > 12 mmHg)
Sample size	388	81
Mean	IAP: 8.254 IBP: 8.351	IAP: 16.173 IBP: 16.455
Variance	IAP: 27.037 IBP: 29.318	IAP: 09.150 IBP: 15.764
Concordance correlation coefficient	0.965	0.903
95% Confidence interval	0.958 to 0.971	0.866 to 0.931
Pearson ρ (precision)	0.966	0.940
Significance level	*P* < 0.0001	*P* < 0.0001
Bias correction factor C_b_ (accuracy)	0.999	0.961

**Fig. 1:**
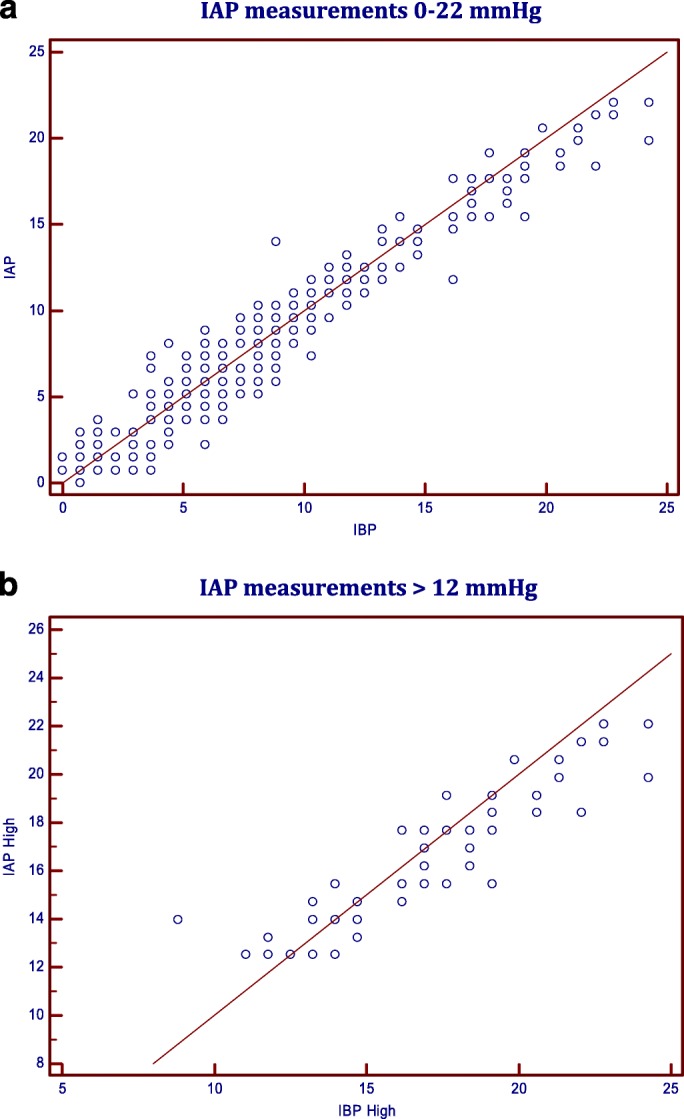
Scatter plot with line of identity for Lin’s Concordance Correlation between the two methods: **a** IAP measurements 0–22 mmHg; **b** IAP measurements > 12 mmHg

Scatter plot diagram with line of identity for the Lin’s concordance correlation between the two methods is depicted in (Fig. 1).

The Bland-Altman analysis indicates that the 95% limits of agreement between the two methods of measurement ranged from − 2.83 to 2.64, the mean of the difference between the two methods (Bias) is − 0.096, and the standard deviation of the bias (precision) is 1.395 (Table 2).

**Table 2:** Bland-Altman Analysis for the two methods: IAP & IBP for all measures (IAP = 0–22 mmHg) Versus measures for High IAP (IAP > 12 mmHg)

First measurement [Method A]	Intra Abdominal Pressure (IAP = 0–22 mmHg)	Intra Abdominal Pressure (IAP > 12 mmHg)
Second measurement [Method B]	Intra Bladder Pressure (IBP with IAP = 0–22 mmHg)	Intra Bladder Pressure (IBP with IAP > 12 mmHg)
Differences		
Sample size	388	81
Arithmetic mean (Bias)	−0.097	−0.282
95% CI	−0.236 to 0.042	−0.619 to 0.056
Standard Error (SE)	0.070829	0.1697
Standard deviation (Precision)	1.3952	1.5278
Coefficient of variation (%)	11.8956	6.6932
Lower limit	−2.8312	−3.2760
95% CI	−3.0694 to −2.5930	−3.856 to − 2.696
Upper limit	2.6379	2.7130
95% CI	2.3997 to 2.8761	2.133 to 3.293

**Fig. 2:**
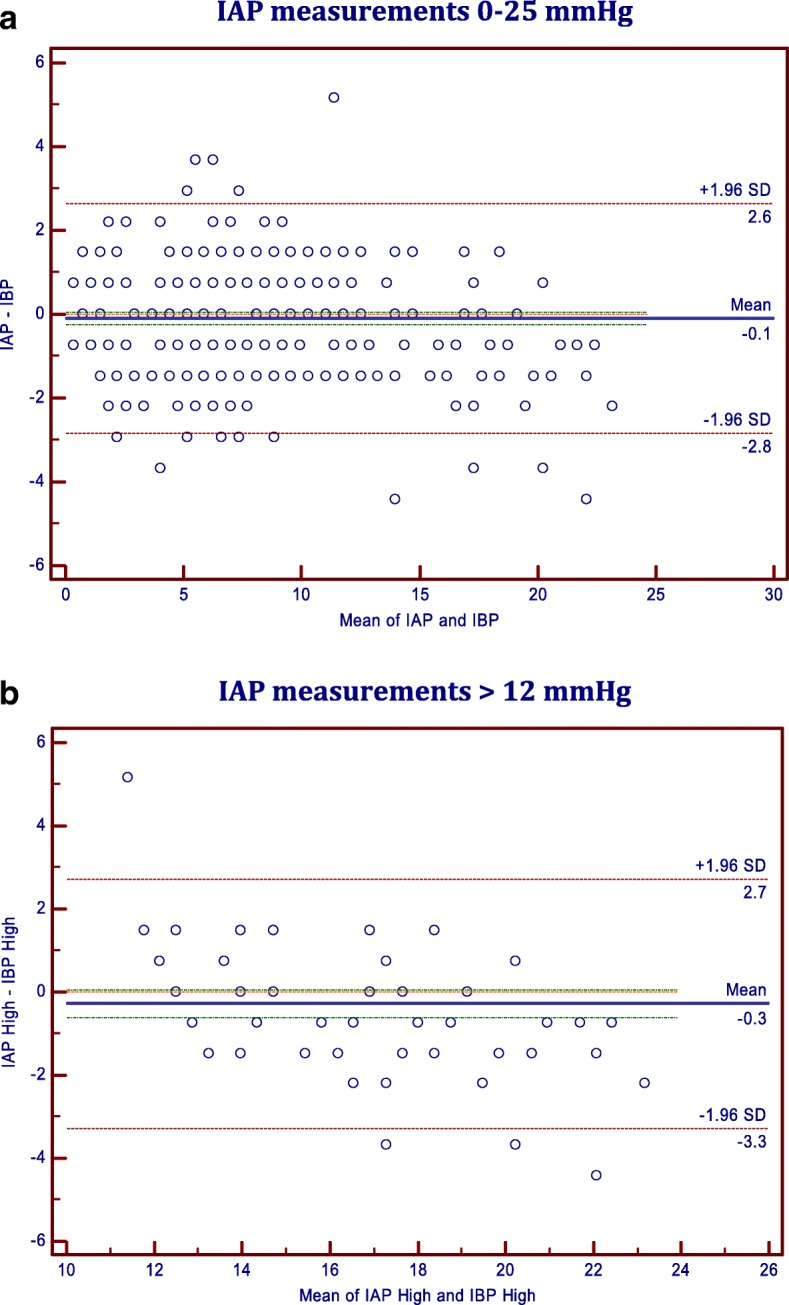
Bland-Altman Scatter Diagram for the two methods: **a** IAP measurements 0–25 mmHg; **b** IAP measurements > 12 mmHg

This indicates that the two methods provide similar measures because the level of agreement lies within the accepted range, both clinically and according to the recommendations of the WSACS [8]. The percentage difference or the coefficient of variation (defined as the precision divided by mean lAP is 11.89%, (should be no higher than 15–20% according to the recommendations of the WSACS [8]). Bland-Altman Scatter Diagram for the difference between the two methods is illustrated in (Fig. 2).

Likewise, Pearson correlation coefficient and Bland-Altman analysis were calculated for IAP greater than 12 mmHg (IAH) (Tables 1 and 2, Figs. 1 and 2). Both showed comparable results that are accepted both clinically and according to the recommendations of the WSACS. The Bland-Altman analysis indicates that the 95% limits of agreement between the two methods of measurement ranged from − 0.62 to 0.05, the mean of the difference between the two methods (Bias) is − 0.281, and the standard deviation of the bias (precision) is 1.395 (Table 2).

Lin’s Concordance correlation coefficient (LCCC) between the two measurements was 0.965 (95% confidence interval: 0.95–0.97) (Table 1). A scatter plot of the measurements with line of identity is represented in (Fig. 1). This shows a “substantial” strength of agreement based on the descriptive scale suggested by McBride for interpreting LCCC [12]. LCCC for high measurements (IAP > 12 mmHg) was 0.90 (95% confidence interval: 0.87–0.93) (Table 1), which indicates a “moderate” strength of agreement based on the descriptive scale suggested by McBride [12]. A scatter plot of the measurements with line of identity is represented in (Fig. 1b). This implies that intra bladder pressure measurements for higher pressures higher than 12 mmHg may be less reliable, according to McBride, for the early detection of intra-abdominal hypertension.

Likewise, Cronbach’s alpha for estimating internal consistency reliability for all measurements including subsets for high (IAP > 12 mmHg) and very high (IAP > 20 mmHg) measurements. Internal consistency reliability was excellent for all measurements as well as high measurements (Cronbach’s alpha = 0.98 and .95 respectively), but only acceptable for very high measurements (Cronbach’s alpha = 0.79) (Table 3), most likely because of the smaller number of measurements (IAP > 20 mmHg, 12/388).

**Table 3:** Cronbach’s Alpha “Estimate of internal consistency” for All measurements: IAP & IBP for all measures (IAP = 0–22 mmHg) Versus measures for High IAP (IAP > 12 mmHg)

		IAP = 0–22 mmHg	IAP > 12–22 mmHg	IAP > 20–22 mmHg
Case Processing Summary	Valid	388	81	12
	Excluded	0	307	376
	Total	388	388	388
Item Statistics	Mean IAP	8.2542	16.1732	21.3312
	Mean IBP	8.3509	16.4547	22.4345
	Standard Deviation IAP	5.19970	3.02496	.54325
	Standard Deviation IBP	5.41462	3.97039	1.23482
Reliability Statistics	Cronbach’s Alpha	.982	.951	.787
	Cronbach’s Alpha Based on Standardized Items	.983	.969	.936

## Discussion

The consequences of increased IAP and ACS are well known and are a source of significant morbidity and mortality in critically ill patients [1, 4, 6]. Therefore, the ability to determine IAP by a simple, reliable and non-invasive technique is of vital importance for the proper management of critically ill patients with IAH, and taking the necessary measures to avoid the occurrence of ACS.

Urinary bladder pressure measurement is the most widely used technique as an indirect method for IAP assessment, but the accuracy and reproducibility are not well established among researchers [5]. Most of their resources come from animal lab studies, the validity of which is questioned because of considerable anatomical differences from humans. One of the major variations is that the bladder being mostly intra-peritoneal in studied animals [2, 6].

Indirect measurements of IAP are position dependent and may not correlate well with the IAP except in the supine position where the mean increment in bladder pressure reflected most closely the IAP [3]. Although not widely accepted, the presence of air-bubbles in the fluid-filled system may partly account for measurement inaccuracy leading to over- or under-estimation [5, 14]. In our study, all pressure measurements were performed with the patients lying in supine position and the pressure transducers zero reference level placed at the symphysis pubis. The system was also carefully checked for air bubbles.

Johna et al. [4] found that IBP did not reflect the actual IAP (limited up to 15 mmHg) during laparoscopic cholecystectomy. They concluded that further research is needed to identify potential variables that may affect the relationship between the urinary bladder and abdominal cavity pressures [4]. Regrettably, they limited their measurements to a maximum of 15 mmHg for the sake of patients’ safety in contrary to the recommendations of the World Society of Abdominal Compartment Syndrome (WSACS) [8]. Moreover, their methodology was criticized [5]. On the other hand, supporting evidence for the reliable use of bladder as an indirect method for measuring IAP came from Yol et al. who compared bladder pressure with insufflator pressure during laparoscopic cholecystectomy in 40 patients. They found a very good correlation between the two measurements (*r* = 0.973, *p* < 0.0001) [7]. Unfortunately, they also limited their measurements to a maximum of 15 mmHg for the sake of patients’ safety, and correlation coefficient was not the appropriate statistical test for comparing two methods of measurement [8, 11]. Fusco et al. [6] measured IBP under different laparoscopic procedures with bladder volumes at 0, 50, 100, 150, and 200 ml at IAP of 0.5, 10, 15, 20, and 25 mmHg. They concluded that IBP closely approximates IAP and that instillation of 50 ml of fluid into the bladder improved the accuracy of the IBP at higher intra-abdominal pressures.

Techniques of simulating increased IAP via instillation of normal saline or insufflation of carbon dioxide as in laparoscopy are considered artificial environments that are far from being ideal, which make it difficult to validate indirect IAP measurements methods and extrapolating them to critically ill patients [15]. Laparoscopy may not reflect the situation of the original pathology that caused IAH, and pressure measurements may not be reliable in patients who have respiratory distress, sepsis with capillary leak, fluid overload, ascites or bladder infection, and may be contraindicated in patients who had bladder surgery or trauma.

Bladder compliance varies within and between patients. Larger instillation volumes may cause overestimation of IAP. Therefore, small volumes to a maximum of 25 ml, enough to create a fluid column and to remove air should be used [16]. The new WSACS guidelines recommend the use of 25 ml of sterile saline in adults and 1 ml/kg (minimum 3 ml – maximum 25 ml) in children [17]. In our study we used the older WSACS recommendation [9] of 50 ml sterile saline to prime the urinary bladder. Our goal was to gradually insufflate CO_2_ intra-peritoneally to approximately 22 mmHg to avoid any deleterious effects on patients’ hemodynamics.

Care must be taken when interpreting the results that compare different methods of measurements. Such methods are occasionally analyzed inappropriately by using correlation coefficients or regression analysis, which are often misleading if used alone. Correlation coefficient measures the strength of a relation (correlation) between two variables, not the agreement between them [8]. According to Bland and Altman [11], “It would be amazing if two methods designed to measure the same quantity were not correlated”. Therefore, the test of significant correlation is irrelevant to the question of agreement. The WSACS recommend the use of Bland-Altman analysis as an alternative approach to assess the limits of agreement between the two methods, that is how much the new method under assessment is likely to differ from the old or standard one [8, 11]. If this is accepted clinically, we can replace the old standard method by the indirect new one. How far apart measurements can be accepted clinically is a question of judgment, which depends on the clinical context, i.e. would the 95% limits of agreement between the two measurement methods meaningfully affect the interpretation of the results? [18]. Good agreement between two methods, as recommended by the WSACS, is defined as follows: the mean difference between the two measurements (bias) does not exceed 1 mmHg; the precision (the standard deviation of the bias) is not greater than2 mmHg and the 95% limits of agreement lies between ± 4 mmHg, and a maximal percentage error of 25% [8]. The percentage difference or the coefficient of variation (defined as the precision divided by mean lAP (= 11.757% in our study) should also be provided, and be no higher than 15–20% [8].

In our study, the Bland-Altman analysis indicates that the mean difference (bias) was − 0.09668, the precision was 1.3952, and the 95% limits of agreement between the two methods ranged from − 2.83 to 2.64. This indicates a high level of agreement between the two methods and we came to the same conclusion, as Fusco et al., that IBP closely approximates IAP.

LCCC between the two measurements was 0.965 (95% confidence interval: 0.958–0.971) (Table 1). This shows a “substantial” strength of agreement based on the descriptive scale suggested by McBride for interpreting LCCC [13]. Internal consistency as demonstrated by Cronbach’s alpha was generally excellent (Cronbach’s alpha = .95–.98). This has a high implication clinically because our aim is to evaluate high-risk patients for the early detection of IAH.

Furthermore, we calculated the correlation coefficient, Lin’s concordance correlation, Cronbach’s alpha and Bland-Altman analysis for IAP higher than 12 mmHg as a subgroup (20.9% of all measurements) (Tables 1, 2 and 3, Figs. 1 and 2). Although it was not as evident in Lin’s concordance correlation [LCCC = 0.90], perhaps because of lack of agreement on the interpretation of LCCC for strength of agreement, Cronbach’s alpha and Bland-Altman method plot showed evidence that Intra bladder pressure measurements for IAH were also reliable for detecting higher levels of IAP. The fact that LCC showed a “moderate” strength of agreement for high readings based on the descriptive scale suggested by McBride [13] makes it less reliable, according to McBride, for the early detection of intra-abdominal hypertension. Although Cronbach’s alpha showed an excellent internal consistency, it was only acceptable (Cronbach’s alpha = .79) for very high pressures (IAP > 20 mmHg, 20.9% of all measurements), probably because of the few measurements taken at that range.

We did not try IAPs higher than 23 mmHg in our study. We felt it would be unethical to jeopardize our patients’ safety for the sake of the study.

There are some limitations to our study. Firstly, we recruited normal subjects, undergoing laparoscopic cholecystectomy, and subjected them to a situation that may resemble IAH. According to the recommendations of the WSACS, patients with at least two risk factors for IAH should be considered for the study [9]. In addition, the new recommendations of WSACS [17], namely the amount of saline instilled into the urinary bladder, the use of mid-axillary line for zero balancing should be taken into consideration. Secondly, in contrary to the relatively slow development of ACS, the time given for each measurement was relatively short due to the rapidity of gas insufflation and the continuous nature of recording the increments in IAP and IBP. Thirdly, the WSACS recommended that at least 50% (later adjusted to one third) of measurements with an elevated IAP (12 mmHg or more), and at least 5% of the measurements with grade III IAH (20–25 mmHg) [9, 10]. In our study, 388 simultaneous pressure measurements from all 21patients were recorded at varying IAPs. Out of these pressure measurements, 20.9% were above 12 mmHg (IAH) and 3.1% were above 20 mmHg (grade III IAH). The relatively infrequent high-pressure recordings above 20 mmHg in our study were due to unintentional abdominal manipulation. Fourthly, pressure measurements were recorded with the patients completely relaxed under general anesthesia patients, irrespective of the phase of respiratory cycle and gastric deflation by a naso-gastric tube. We assumed that any change in IAP caused by increased intra-thoracic or intra-gastric pressures would be accompanied by a synchronous increase in both IAP and IBP. This view is supported by Yol et al. [8], who found that when air was infused into the stomach via a nasogastric tube, the IAP and IBP increased simultaneously. Likewise, when the stomach was deflated, the two pressures decreased simultaneously to the pre-insufflation levels. Finally, Patients with higher body mass index (BMI) have higher resting intra-abdominal pressure which may stress the bladder and pelvic floor. Although there is a positive correlation between IAP and BMI, we assumed that it will affect both intra-abdominal and intra-bladder pressures equally. Nevertheless, BMI could be a confounding factor that was not considered in our study.

## Conclusion

Although not fully compliant with WSACS recommendations, our study showed that IBP measurement is a simple, minimally invasive method that may reliably estimates IAP in patients placed in supine position. Measurements for pressures higher than 12 mmHg may be less reliable. When applied clinically, this should alert the clinician to take safety measures to avoid abdominal compartment syndrome (ACS).

## Highlights

Intra-abdominal Hypertension (IAH) is defined as an intra-abdominal pressure (IAP) equal to or above 12 mmHg and abdominal compartment syndrome (ACS) is defined as an IAP above 20 mmHg with evidence of organ dysfunction/failure. Clinically, significant intra-abdominal hypertension may be present in the absence of abdominal distension. Increased IAP is commonly measured by recording urinary bladder pressure. This study shows that urinary bladder pressure measurement is a simple, minimally invasive method that reliably estimates IAP. Urinary bladder pressure measurement can be used clinically to evaluate high-risk patients for the early detection of IAH and alert the clinician to take safety measures to avoid abdominal compartment syndrome (ACS).

## Abbreviations

ACS: Abdominal compartment syndrome
BMI: Body mass index
CO_2_: Carbon dioxide
IAH: Intra-abdominal hypertension
IAP: Intra-abdominal pressure
IBP: Intra-bladder pressure
LCCC: Lin’ concordance correlation coefficient
SPSS: Statistical package for the social sciences 20
WSACS: The World Society of Abdominal Compartment Syndrome
